# HIV Persistence, Latency, and Cure Approaches: Where Are We Now?

**DOI:** 10.3390/v16071163

**Published:** 2024-07-19

**Authors:** Tessa C. Chou, Nishad S. Maggirwar, Matthew D. Marsden

**Affiliations:** 1 Department of Microbiology and Molecular Genetics, School of Medicine, University of California, Irvine, CA 92617, USA; tessac@uci.edu (T.C.C.); nmaggirw@uci.edu (N.S.M.); 2 Department of Medicine, Division of Infectious Disease, School of Medicine, University of California, Irvine, CA 92617, USA

**Keywords:** HIV, cure, latency, reservoir, persistence, animal model

## Abstract

The latent reservoir remains a major roadblock to curing human immunodeficiency virus (HIV) infection. Currently available antiretroviral therapy (ART) can suppress active HIV replication, reduce viral loads to undetectable levels, and halt disease progression. However, antiretroviral drugs are unable to target cells that are latently infected with HIV, which can seed viral rebound if ART is stopped. Consequently, a major focus of the field is to study the latent viral reservoir and develop safe and effective methods to eliminate it. Here, we provide an overview of the major mechanisms governing the establishment and maintenance of HIV latency, the key challenges posed by latent reservoirs, small animal models utilized to study HIV latency, and contemporary cure approaches. We also discuss ongoing efforts to apply these approaches in combination, with the goal of achieving a safe, effective, and scalable cure for HIV that can be extended to the tens of millions of people with HIV worldwide.

## 1. Background

**Figure 1:**
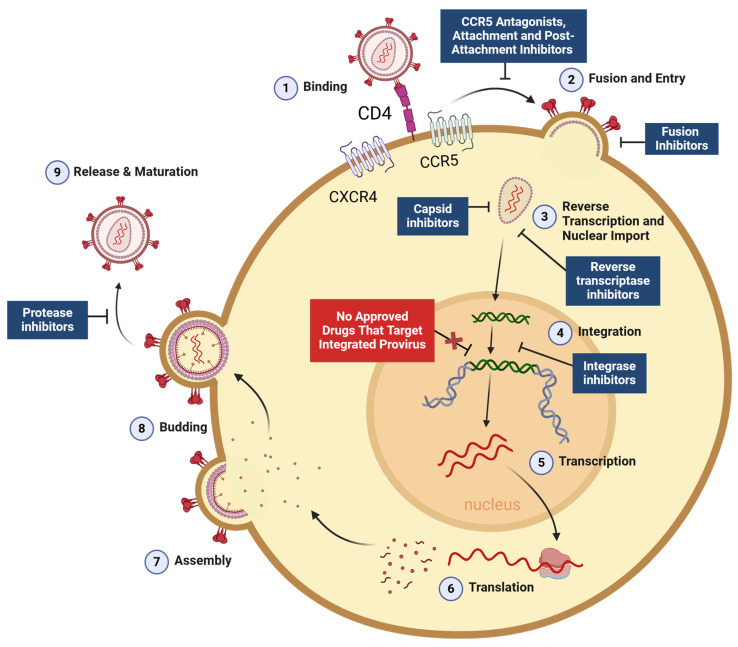
**Steps of the HIV life cycle targeted by currently available antiretroviral drugs**. HIV binds to the cellular receptor CD4 and a co-receptor (typically CCR5 or CXCR4) to enter a target cell through interaction with the viral gp120 envelope protein. Upon binding, the envelope transmembrane protein gp41 facilitates virus–host cell membrane fusion, triggering release of the viral core into the cytoplasm. A double-stranded DNA copy of the viral RNA is then reverse transcribed around the time it is shuttled into the nucleus, then the HIV integrase enzyme permanently inserts the HIV genome into the host-cell chromosomal DNA. Now stably integrated, the HIV provirus is transcribed into RNA, which is exported to the cytoplasm (in some cases after splicing). After the RNA is translated into proteins, immature virions are assembled and bud through the plasma membrane. The HIV protease enzyme catalyzes virion maturation by cleaving polypeptides within the new virion. Currently approved classes of antiretroviral drugs are shown in blue boxes. HIV latency occurs when the provirus pauses after integration, during which time it expresses little or no RNA and no viral proteins. As there are no currently approved antiretroviral drugs that target an integrated HIV provirus, latently infected cells can persist despite ART (red box).

Human immunodeficiency virus (HIV) remains a leading global health concern. In 2022, 39 million people across the world were living with HIV, 1.3 million people were newly infected that year, and over 40 million people had died of acquired immunodeficiency syndrome (AIDS) since the start of the HIV epidemic [1]. Combination antiretroviral therapy (ART) medications are used to prevent HIV acquisition and treat HIV infection [2]. These medications target different steps of the HIV life cycle to prevent virus replication. An impressive array of different antiretroviral drug classes have advanced to clinical use for HIV treatment, including pre-attachment, post-attachment, and fusion inhibitors, capsid inhibitors, non-nucleoside reverse-transcriptase inhibitors, nucleoside and nucleotide reverse-transcriptase inhibitors, integrase strand transfer inhibitors, and HIV protease inhibitors (Figure 1). During optimal treatment, several different antiretroviral drugs (typically three) belonging to at least two different classes are provided in combination to effectively suppress virus replication and reduce the probability of drug-resistant virus emerging in the treated individual [2]. Ideally, this treatment will reduce plasma viral loads to undetectable levels and prevent HIV infection from advancing to AIDS. ART is therefore critical in extending the lifespan of people with HIV (PWH) and has saved millions of lives. However, ART is not a cure for HIV infection. Though it successfully inhibits the replicating virus, ART is unable to target stable reservoirs of HIV, including within latently infected CD4^+^ T cells [3,4,5,6], which persist in the host despite long-term ART.

**Figure 2:**
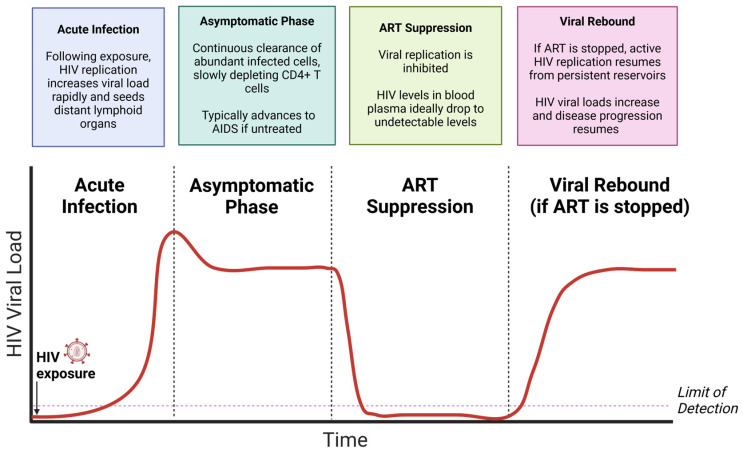
**HIV plasma viral loads at different phases of HIV infection**. Upon acquisition of HIV, viral loads rapidly increase. After initial viremia is reduced by the adaptive immune response (including HIV-specific CD8^+^ T cells), infection proceeds to a long and typically asymptomatic period that often lasts 10 years. In this stage, very high levels of virus are produced each day leading to high plasma viral loads, but this is offset by the immune response and regeneration of immune cells by the hematopoietic system. If left untreated, infection will eventually progress to AIDS. This occurs when CD4^+^ T cell numbers are significantly reduced (<200 cells/microliter blood), and the virus has caused sufficient immunological damage so that efficient immune responses can no longer be mounted. The immunodeficient individual is then vulnerable to many opportunistic infections and diseases. Upon initiating ART treatment, HIV viral loads in plasma are ideally reduced to undetectable levels. For HIV to remain suppressed, ART must be continually taken to prevent viral rebound from latent reservoirs that are not cleared by currently available ART.

Replication-competent latent HIV reservoirs are formed when an infected cell harbors an intact, integrated HIV provirus that is expressing little or no RNA and no viral proteins but can be induced to produce new infectious virions with appropriate cellular stimuli [3,5,6,7]. Due to the long-lived nature of memory CD4^+^ T cells, integrated HIV can lie dormant as a provirus for decades, undetected by the immune system until reactivation [8,9,10]. Consequently, if ART treatment is stopped then viral outgrowth from reservoirs followed by rapid replication (virologic rebound) invariably occurs in PWH (Figure 2). ART must therefore be taken for life to continually suppress viral replication. ART has other important limitations, including a lack of drug availability in some parts of the world, and requirements for a robust medical infrastructure to deliver therapy and monitor patients for continued virus suppression, which is not available in many resource-limited settings [1]. Additional problems with current therapies include, but are not limited to, the development of antiviral drug resistance [11], side effects associated with long-term drug treatment and ART toxicity, ongoing immune activation and immunological dysfunction in ART-treated individuals [12,13], ART regiment adherence, and continued social stigma faced by PWH. Hence, there remains a compelling need to develop a cure for HIV to permanently prevent rebound from occurring and remove the requirement for long-term ART. To achieve this important goal, further characterization of the latent HIV reservoir is needed, along with the development of new methods to eliminate persistent viral reservoirs.

In this review, we describe the establishment of latent HIV reservoirs and the major challenges associated with eliminating them, highlight current small-animal models that are used to study HIV persistence, and illustrate promising contemporary strategies that are being advanced to eradicate the latent reservoir.

## 2. Establishment and Maintenance of HIV Latency

Lifelong ART is critical for preventing disease progression in PWH. In the event ART is stopped, even many years after therapy initiation, the virus rapidly re-emerges from rare reservoir cells and replicates to high levels. Understanding the nature of the viral “reservoir” that maintains infectious HIV genomes through long-term ART and eliminating sources of rebounding virus is, therefore, a major focus of the HIV field. HIV infects several different cell types, including CD4^+^ T helper cells and macrophages [14]. As macrophages are more resistant to the cytopathic effects of HIV infection, they may harbor low levels of HIV expression, thus potentially supporting chronic infection and persistence in immunoprivileged sites. However, there is increasing evidence that macrophage-lineage cells can support latent infection with HIV and may serve as viral reservoirs [15,16,17,18]. Yet, the best-defined and probably the largest persistent HIV reservoir consists of latently infected CD4^+^ T cells [3,4,5], which contain latent HIV genomes in the form of stable, [3,10] replication-competent proviruses integrated in host chromosomes present for the duration of the host cell’s life. As these cells are not expressing viral proteins, they are not recognized as infected by the host immune system and can thus evade clearance by immune effector mechanisms.

A crucial factor that influences HIV reservoir characteristics is the distinctive physiological properties of the preferred host cells for HIV in vivo. T cells, including CD4^+^ T cells, can exist in multiple different metabolic or activation states and, importantly, can transition between these states during the lifespan of the cell. While quiescent and waiting to encounter their cognate antigen, T cells are metabolically inactive, rarely divide, and are highly refractory to HIV infection [19]. Once stimulated by cognate antigen recognition, they enter a metabolically active state and proliferate, readily supporting HIV replication and producing abundant progeny virions. Though highly activated CD4^+^ T cells can harbor latent HIV genomes [20], latently infected cells are typically quiescent. Multiple elements make activated CD4^+^ T cells the preferred hosts for efficient HIV replication over their resting counterparts. Prior to integration, these include comparatively low CCR5 co-receptor expression on resting cells [21] (although unstimulated cells do also express CXCR4) and inefficient reverse transcription in non-activated cells [19], perturbed through abundantly expressed SAMHD1, which hydrolyzes the dNTPs necessary for HIV cDNA synthesis mediated by reverse transcriptase (RT) [22]. Hence, it is likely that most latently infected cells originally became infected by HIV when the host cell was partially stimulated with cytokines [23], other stimuli, or fully activated through their T-cell receptor and then reverted to a resting state before they could be killed by virus cytopathic effects or immune effector mechanisms. This could occur, for example, when an infected CD4^+^ T cell transitions to a resting memory cell as part of the normal process of immunological memory formation after antigen exposure. Indeed, CD4^+^ T cells infected during the process of transitioning from effector to memory cells may be more likely to form post-integration latency due to increases in CCR5 expression coupled with downregulation of cellular transcription [24]. If the resting latently infected host cell is subsequently activated, viral expression can be induced, resulting in the production of new infectious virions capable of seeding rebound.

Several different molecular mechanisms have been identified that can reversibly inhibit expression of HIV and thereby contribute to latency. These include transcription factor availability, the provirus integration site, epigenetic modifications, and the efficiency of transcriptional elongation [25,26,27]. These mechanisms can act individually or in concert to inhibit virus expression, making HIV latency regulation complex, multifactorial, and challenging to reliably disrupt.

### 2.1. Transcription Factor Availability

Following integration, the limited presence of host transcription factors in the nucleus of resting CD4^+^ T cells significantly hampers HIV RNA expression, as they are critical for efficient HIV long terminal repeat (LTR)-mediated transcription. The 5′ LTR promoter possesses multiple transcription factor binding sites that positively regulate virus expression, notably nuclear factor-kappa B (NF-kB) and nuclear factor of activated T cells (NFAT) [28,29]. NF-kB and NFAT are sequestered in the cytoplasm in resting cells, but translocate into the nucleus upon cellular activation, where they can promote HIV transcription. NF-kB is present in the cytoplasm of resting cells as a p50/RelA heterodimer, which is associated with I-kB and thereby held in an inactive form. Meanwhile, p50/p50 homodimers of NF-kB, which lack the transactivation domain of the active NF-kB p50/RelA heterodimer, are bound to the proviral HIV LTR in the nucleus of these resting cells. Upon cellular activation, I-kB is phosphorylated, ubiquitylated, and degraded by the 26S proteasome, which allows the p50/RelA heterodimer to translocate to the nucleus and bind to the 5′ LTR promoter, displacing the inactive p50/p50 homodimers. NFAT is similarly present in the cytoplasm of resting cells, and is dephosphorylated upon T cell activation, which induces its nuclear localization [27]. NFAT or NF-kB p50/RelA heterodimers bound to the 5′LTR induce transcriptional activation by recruiting histone acetyltransferases, including p300/CBP, which cause acetylation in histone tails [25,27,30,31]. Expression of the provirus by NF-kB is also dependent on protein–protein interaction between NF-kB and Sp1, which is constitutively bound to an adjacent site on the LTR promoter [32].

### 2.2. Integration Sites

While HIV is capable of integrating throughout the human genome, transcriptional profiling of CD4^+^ T cells indicates that the process is not random [33,34]. HIV preferentially integrates into sites in the host cell genome near active transcriptional units [34,35,36] and regional hotspots of enriched active genes near the nuclear envelope, with infected resting CD4^+^ T cells actively expressing the majority of genes containing integration sites [33]. This bias towards integrating in active genes improves the chances of successful expression, though integration-site frequency may vary in different populations of PWH. Notably, elite controllers have been observed to have increased proviral integration in repressed heterochromatin [37], which primarily consists of satellite DNA. However, the HIV provirus can also be influenced by host genes surrounding the integration site. Transcriptional interference can occur when transcription from the upstream promoter is ongoing as RNA polymerase II (RNAPII) reaches a downstream HIV provirus in the same orientation, leading to HIV-1 promoter occlusion and silencing of transcription [38] in some model systems and, conversely, enhancement of expression in others [39]. Alternatively, in the event HIV integrates in an opposing orientation of an actively transcribed gene, the opposing action of the convergent promotors has an inhibitory effect on HIV transcription and expression [39]. Notably, the overall contribution of transcriptional interference to HIV transcript expression is complex and has been observed to vary widely in different cell line models of HIV latency and primary cell assays [40].

### 2.3. Epigenetic Modifications

Following integration, HIV DNA is incorporated into the host genome, and, like all genes, is condensed into chromatin. RNA polymerase-mediated transcription and production of full-length HIV transcripts requires accessible chromatin, dictated by whether it is in an active or silent state. As such, methylation, ubiquitylation, and epigenetic chromatin modifiers can affect gene expression. Epigenetic chromatin modifiers regulate chromatin blocks and the transition between “loose” euchromatin and “compact” heterochromatin, and play key roles in regulating latency [41,42,43,44]. More specifically, histone acetylation and deacetylation are well understood as epigenetic regulators of HIV latency, due to histone deacetylase (HDAC)-mediated histone acetylation and the subsequent chromatin “tightening” being correlated with transcriptional repression, whereas histone acetyltransferases (HATs) unwind chromatin and enable transcriptional activation via acetylation [42,45].

Histone modifications additionally have important implications for nucleosome positioning by affecting the association between nucleosomes and DNA, thus potentially restricting transcription [46]. In the context of HIV latency, the deacetylation of viral nucleosome nuc-1 has been shown to be associated with LTR repression, indicating chromatin structure may dictate whether HIV is expressed [42]. Nuc-1 thus is also subject to chromatin remodeling complexes such as BAF (Brg1/Brm-associated factor), which can alter nuc-1 positioning on the LTR into a less energetically favorable position, promoting transcription repression [44].

### 2.4. Transcriptional Elongation

Given its role as a transcriptional activator, the presence of HIV Tat and its associated cofactors is critical for efficient HIV expression. Before Tat is expressed, HIV transcription is inefficient and associated with promoter-proximal pausing of RNA transcription and deficits in nascent RNA elongation [47]. However, during productive HIV replication, a small amount of Tat protein is still expressed, which binds to the transactivation response element (TAR) RNA stem loop present at the 5′ end of all HIV transcripts [48,49,50,51,52,53,54]. Tat enhances HIV transcription through several mechanisms, including recruitment of elongation regulator P-TEFb to the nascent viral RNA, which relieves the transcriptional block and enables rapid efficient full-length HIV transcript expression [55]. Factors that contribute to HIV latency in resting CD4^+^ T cells include very low levels of P-TEFb in these cells, and a lack of sufficient Tat expression in the absence of T cell activation [25].

## 3. Challenges of HIV Latency: Why Is the Latent Reservoir Difficult to Eliminate?

Several characteristics of the latent reservoir make it particularly challenging to study and eliminate. Latently infected cells typically express little or no viral RNA and no viral proteins, and no reliable marker has been identified that externally distinguishes these cells from their non-infected counterparts. Resting memory CD4^+^ T cells harboring integrated HIV are also rare in vivo, with an occurrence of approximately one in every million resting CD4^+^ T cells [56], typically translating to around a million latently infected cells per patient. However, latently infected-cell frequencies and overall reservoir size also vary substantially from person to person [56]. Moreover, this estimate of reservoir size is based on outgrowth of infectious HIV following a single round of ex vivo stimulation, and the total size of the reservoir based on cells harboring apparently intact HIV proviral sequences can be substantially higher [20].

Latently infected cells are also widely distributed throughout the body. Around 2% of CD4^+^ T cells are in the peripheral blood, but most are present in tissues [57], making the majority of the reservoir difficult to access and study in PWH. Simultaneously, the reservoir decays slowly during ART (if at all), and has been documented to have an initial half-life of around 44 months in the first few years of therapy [10] with no long-term reduction observed even after over two decades of treatment [10]. Latency is also established very early in the course of infection, typically within the first days or weeks following exposure [58,59]. Hence, the latent reservoir shows remarkable stability, and is sufficient to maintain lifelong infection, even with continuous ART.

### 3.1. Clonal Expansion and Homeostatic Proliferation

Several mechanisms can contribute to the maintenance of the latent reservoir over extended periods of time. These include the naturally long lifespan of memory CD4^+^ T cells in vivo and their propensity to divide via homeostatic proliferation [60,61] or antigen-driven clonal expansion [60,61].

Immunological memory is maintained in part by the occasional homeostatic proliferation of memory T cells, driven by cytokines including interleukin-7 (IL-7) [62]. This cell division can, in some cases, lead to expansion of cell clones containing a latent HIV provirus, producing many cell clones containing genetically identical HIV proviruses with the same HIV integration site [61,63]. Alternatively, the reservoir can be maintained through antigen-dependent clonal expansion. Upon recognizing their cognate antigen, memory CD4^+^ T cells divide. If a cell possesses an integrated latent HIV provirus and undergoes antigen-dependent clonal expansion, it will generate identical clones which contain integrated latent proviruses, enabling the latent reservoir to persist and potentially expand. Latently infected T cells specific to antigens, including cytomegalovirus (CMV), have been identified to possess identical proviral sequences [62], suggesting responses to common antigens including those encoded by other viruses [64,65] may also induce clonal expansion of the latent reservoir.

In some cases, the specific integration site of the HIV provirus may also influence the host cell in a way that increases propagation of that infected clone [63]. In the event the provirus is integrated into certain genes in the same orientation as the host gene transcription, the resulting insertional mutagenesis may induce proliferation of HIV-infected CD4^+^ memory T cells while dysregulating cellular growth. For example, identical clones of proviruses in STAT5B, MKL1, and BACH2 have been detected in PWH on ART with a broad tissue distribution [63,66].

Recent estimates suggest that more than 50% of all latently infected cells after ART treatment result from some type of clonal expansion [67]. While host cell activation and proliferation can be associated with latent HIV reactivation and killing of the host cell, these studies illustrate that division of a cell harboring an integrated latent HIV provirus does not always lead to the death of that host cell. Instead, host cell proliferation can function as a mechanism for reservoir expansion, potentially accompanied by the selection of cellular clones bearing proviruses that do not reactivate or result in the killing of the host cell through viral cytopathic effects during cell division.

### 3.2. Proviral Genetic Diversity and Its Functional Consequences

Beyond the diverse sites of latent HIV integration within the human genome, the HIV provirus itself has high genetic diversity, in part due to the error-prone nature of the reverse-transcriptase (RT) enzyme. RT is a low-fidelity enzyme that lacks exonuclease proofreading activity, resulting in frequent mutations. Moreover, during chronic HIV infection, virus replication occurs simultaneously in many millions of cells each day. The HIV genome is also very tolerant to mutations and can continue producing infectious virus with mutations present at many different sites throughout the genome. Together, these factors allow HIV to evolve rapidly—one million times faster than mammalian DNA [68]. This large diversity provides an abundant pool of mutants for natural selection to act upon. Strong selection pressures may thus favor growth of viruses with resistance to ART drugs, or those bearing CTL escape mutations [69,70,71] or mutations conferring resistance to antibodies [72,73]. These mutant viruses can be deposited in the latent reservoir and maintained during ART, making the reservoir an important repository of clinically relevant mutant viruses.

### 3.3. Complications of Defective Proviruses

Errors during reverse transcription also may lead to the generation of integrated proviruses with mutations that prevent the production of fully infectious progeny virions. A significant subset of these “defective proviruses” may not be able to express at all, but others have been shown to encode and produce viral proteins such as Gag and/or Nef [74]. These proteins can be recognized by the host immune system, enabling CTL-mediated killing of the host cell [75], contributing to persistent immune activation and chronic inflammation in PWH in ART [76]. The presence of these defective proviruses also complicates molecular analysis of replication-competent latent virus using assays that detect short subgenomic HIV sequences, as the analyzed sequences may not be derived from intact proviruses [77]. Given the role defective proviruses may play in obscuring reservoir size and driving persistent immune activation in PWH on ART, it remains important to understand the factors dictating proviral transcription and protein production for improved therapeutics, as current ART treatments may be unable to target chronic inflammation resulting from HIV RNA produced by defective proviruses.

### 3.4. Other Reservoir Cells

Beyond CD4^+^ T cells, other cell types might also serve as viral reservoirs, including myeloid cells [15,16]. In particular, macrophages are readily infected by HIV [78] and tissue resident macrophages such as microglia in the brain can be long-lived [79,80], potentially providing a stable reservoir site. HIV has been detected in macrophages isolated from various tissues in PWH, including the brain and CNS [81,82]. In one recent study, brain myeloid cells were isolated from non-human primates and PWH on ART, and integrated SIV and HIV DNA was detectable in these cells [83]. Virus isolated following ex vivo HDACi stimulation was then able to successfully replicate, supporting the existence of macrophage HIV reservoirs in vivo [83].

In additional work, quantitative viral outgrowth assays with monocyte-derived macrophages [17] were used to show that intact proviruses in macrophages are present in some PWH and capable of producing replication-competent virus that can infect new cells. Moreover, additional cell types have been found in some studies to serve as latent HIV reservoirs and may play a role in pathogenesis [84,85]. Hence, understanding the mechanisms governing HIV latency across all reservoir cell types remains critical for effective cure approaches that target all reservoirs.

In summary, the latent reservoir is dynamic and continuously maintained over time through multiple mechanisms of clonal expansion. Infected cells are widely distributed throughout the body [57,86,87,88], with the majority being concentrated in difficult-to-sample lymphoid organs. Simultaneously, the high genetic diversity of HIV enables its rapid evolution, but errors in viral replication lead to defective proviruses that complicate analysis of the rebound-competent reservoir. As a result of these factors, it remains challenging to identify, isolate, and study latently infected cells.

## 4. Animal Models of HIV Latency

In vitro models of latent HIV infection using cell lines and primary cells have provided useful isolated environments for mechanistic studies of HIV biology and cure approaches, but do not fully recapitulate additional complex host processes in the whole host organism. For example, in vivo circulation, compartmentalization, metabolism, and physiologic processes coordinated by multiple cell and tissue types cannot be adequately modeled solely with in vitro models. Latent reservoirs are also rare in vivo, and primarily reside in difficult-to-access tissues, complicating their study using clinical samples. Furthermore, interventions to deplete the latent reservoir are often experimental in nature and require preclinical in vivo testing before evaluation in people with HIV. Together, these factors necessitate the use of in vivo animal models, which complement and validate in vitro studies while forming foundations for clinical research. Currently, the most commonly used in vivo models of HIV infection include non-human primates (NHPs) and humanized mice.

### 4.1. Non-Human Primate Models

NHP models historically include, but are not limited to, HIV-infected chimpanzees (discontinued for ethical reasons, including their endangered-species status) and rhesus, pigtail, or cynomolgus macaques infected with simian immunodeficiency virus (SIV) or recombinant simian–human immunodeficiency virus (SHIV) [89,90]. Because NHPs share a close phylogenetic relationship with humans and can be infected with related SIV and SHIV, their similar physiology and immune system provide key advantages in accurately modeling natural routes of transmission and HIV pathologies such as chronic immune activation and CD4^+^ T cell depletion. NHP models can be studied in a controlled setting, regulating factors including strain and dose of virus and experimental agents, exposure route, timing of infection, and ART duration, which are otherwise difficult or impossible to control in humans [89,90]. Simultaneously, upon necropsy, they can provide a complete set of viable tissue samples for characterization of the latent reservoir, which are typically unavailable in clinical human studies.

Some limitations of NHP models include their relatively high cost, logistical issues with large-animal housing and handling, species-specific differences between humans and non-human primates, and virologic differences between SIV and HIV. Nevertheless, NHP studies have provided insights into a broad array of topics including viral latency and reservoir analysis [91,92] protection studies [93,94,95], and cure approaches [96,97]. As such, they have proven to be versatile and important in vivo models for HIV persistence during ART.

### 4.2. Humanized Mouse Models

Though there are no known exogenous lentiviruses that can infect non-modified mice, mouse models have been created that allow infection with HIV while supporting the establishment of a latent reservoir, which serve as important in vivo models for use in HIV persistence studies [97,98,99,100,101,102,103,104]. “Humanized mice” are typically used as models for infection with HIV. These are generated by transplanting human cells and/or tissues into suitable recipient mice [105,106,107]. Many different humanized mouse models exist, which vary in the recipient mouse strain used, the human cell or tissue types transplanted, and the specifics of the experimental procedures (reviewed in [105,106,107]). However, the overall goal of these models is to provide a suitable in vivo environment for HIV replication and study how the virus interacts with cells and tissues in a living host.

To prevent transplanted human tissues from being eliminated by innate and adaptive murine immune responses, immunodeficient mouse strains are used as recipients for human cells. Original recipient mice included nude mice that lack T cells due to Foxn1^mu^ mutations [108] and severe combined immunodeficiency (SCID) mice, which possess mutations in in the protein kinase, DNA-activated, catalytic subunit (Prkdc) gene, abolishing both B and T cells [109]. An alternative pathway for interfering with T cell and B cell development is to disrupt one of the recombination activating genes (Rag1 and Rag2), as they are critically important for B and T cell receptor rearrangements [110,111,112,113]. Rag mutations also do not induce the same higher susceptibility to radiation damage that is associated with the SCID mutation, due to its involvement in DNA repair.

Further reductions in immune competency have been achieved by eliminating receptors for key cytokines. The interleukin-2 (IL-2) common γ chain (IL2Rγ) is a component of the receptors for the cytokines IL-2, IL-4, IL-7, IL-9, IL-15, and IL-21 [114,115]. As such, recipient mouse strains with disrupted IL2Rγ do not respond appropriately to these cytokines. When combined with SCID or Rag mutations, this results in a profoundly immunodeficient animal, with the lack of IL-15 in particular contributing to an absence of NK cells in these animals [116]. Mouse strains bearing these common gamma chain mutations include the NOD.Cg-Prkdc^scid^Il2rg^tm1Wjl^/SzJ (NSG), NOD.Cg-Rag1^tm1Mom^ Il2rg^tm1Wjl^/SzJ (NRG), NOD.Cg-*Prkdc^scid^Il2rg^tm1Sug^*/ShiJic (NOG), and C;129S4-*Rag2^tm1.1Flv^ Il2rg^tm1.1Flv^*/J (BRG) mice.

The earliest humanized mouse models for HIV infection included human peripheral-blood leukocyte (hu-PBL) mice [117,118,119]. Hu-PBLs are typically generated through intraperitoneal, intravenous, or intrasplenic injection of mature PBMCs/specific immune cell types into SCID or NSG mice [117]. Despite being a simple, cost-effective model with circulating human immune cells, hu-PBLs are prone to GVHD-induced immune activation [120] occurring within weeks after transplantation, which limits their use in long-term HIV reservoir studies.

SCID-human thymus/liver (hu-Thy/Liv) and human hematopoietic stem cell (hu-HSC or hu-CD34) mice are models where stem cells undergo in vivo differentiation into mature immune cells that can support HIV replication. SCID-hu-Thy/Liv models are composed of a human fetal liver and thymus-derived implant, which is surgically placed under the kidney capsule and provides an environment for CD34^+^ HSCs present in the fetal liver tissue to differentiate into T cells [121]. This generates an organoid that is structurally and functionally similar to a human thymus, but the resultant cells are primarily immature or naïve T cells constrained to the implant tissue, with few circulating human cells. [106]. Hu-HSCs/hu-CD34s are created by making space in the bone marrow for transplanted stem cells (e.g., through irradiation), then injecting CD34^+^ HSCs, which in turn differentiate into a diverse population of human immune cells [122] that can circulate systemically.

Interestingly, NOD/SCID mice injected with CD34^+^ HSCs have been shown to lack peripheral T cells and can support replication of macrophage-tropic HIV, resulting in myeloid-only (MoM) humanized mouse HIV models [16,123], allowing the study of macrophage infection without the confounding presence of T cells. Improved T cell development and mucosal immune cell reconstitution has been achieved by the simultaneous transfusion with CD34+ cells and transplant of Thy/Liv tissue to create the bone marrow–liver–thymus (BLT) mouse [124,125,126]. BLT mice can produce a near-complete complement of human immune lineages in vivo, including T cells, B cells, NK cells, monocyte/macrophages and dendritic cells.

**Figure 3:**
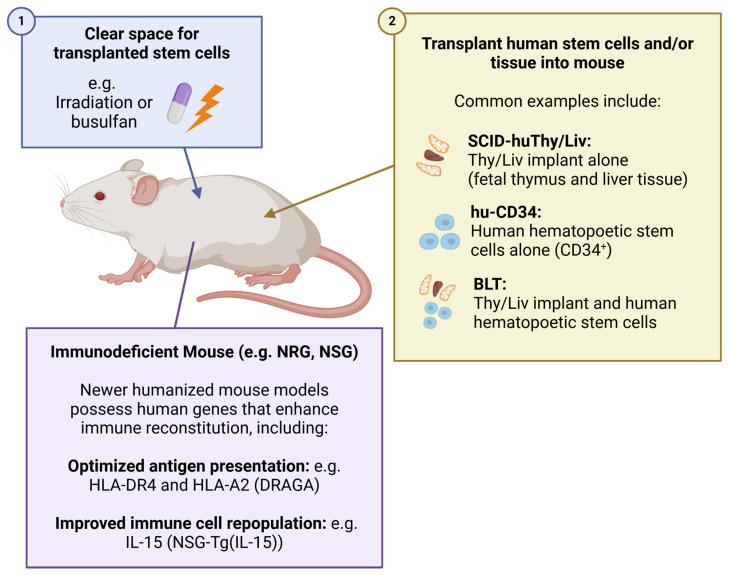
**Schematic of common humanized mouse models for HIV infection and persistence**. HIV does not infect non-modified mice, so common murine models for studying HIV replication, pathogenesis, and cure approaches rely on the use of immunodeficient mice transplanted with human cells and/or tissues (humanized mice). For production of mice with near-complete immune systems, this has historically involved highly immunodeficient recipient mouse strains including the NOD-SCID-common gamma (NSG) or NOD-rag-gamma (NRG), which are either irradiated or treated with busulfan to clear space in the bone marrow, followed by infusion with hematopoietic stem cells. This may be performed alone (hu-CD34) or, in the case of bone marrow/liver/thymus (BLT), performed in conjunction with the transplant of fetal liver and thymus tissue under the kidney capsule to generate a human thymus organoid where T cells can develop on human thymic stroma. More recent advances include the use of recipient mice bearing various important human immune genes that further improve the recapitulation of a working human immune system. For example, DRAGA mice (base strain NRG) expressing human HLAs (allowing optimized antigen presentation) or mice that augment human immune cell repopulation, such as NSG-Tg(IL-15) mice that produce human IL-15, allowing the improved development of NK cells.

While these models allow multilineage immune reconstitutions with different human immune cell lineages, they do have limitations [107], including reduced cell numbers in certain immune lineages and adaptive immune responses that are not as robust as those occurring in humans. This is being addressed with further advances in recipient mouse strains, which are engineered to express human cytokines, chemokines, or leukocyte antigens (HLAs) to facilitate the differentiation of specific immune-cell lineages, or support the production of adaptive T cell and antibody responses (Figure 3).

Examples of such recipient mouse strains include mice expressing either human IL-15 (NSG-Tg(Hu-IL-15)) alone [127,128] or with human signal regulatory protein α (SIRPA) [129], which facilitate reconstitution with human NK cells, and NRG mice expressing HLA—DR4 alone (DRAG) [130] or in tandem with HLA-A2 (DRAGA), which support improved T and B cell function and development upon HSC engraftment [131,132]. NSG-Tg(Hu-IL-15)) mice have been used in recent HIV cure studies, including NK augmentation and bispecific antibody research [133], while HIV-specific antibody responses and viral replication have been characterized in DRAGA mice [134]. In summary, humanized mice are powerful tools for studying HIV infection in vivo, and recent improvements have further enhanced their physiologic relevancy, providing opportunities to more accurately model HIV infection and the HIV–human immune system interaction in vivo.

## 5. HIV Cure Approaches

**Figure 4:**
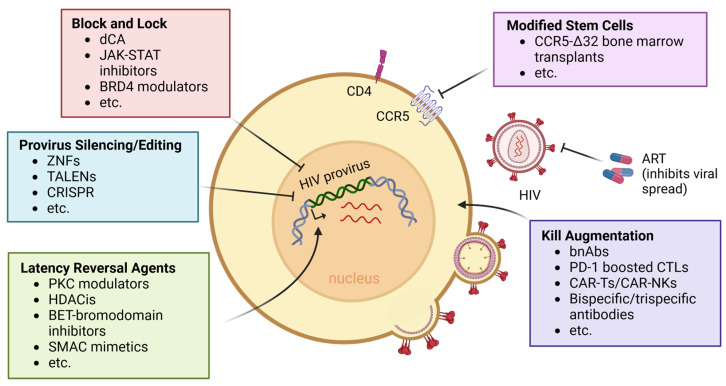
**Current HIV cure approaches**. General approaches to developing a cure for HIV that are currently being explored within the field include block and lock, provirus editing/silencing, latency reversal and kill augmentation (kick and kill), and stem cell transplantation/gene therapy. Block and lock agents include didehydro-cortistatin A (dCA), JAK-STAT inhibitors, and BRD4 modulators. Common provirus editing/silencing approaches have utilized zinc nuclease fingers (ZNFs), transcription activator-like effector nucleases (TALENs), and clustered regularly interspaced short palindromic repeats (CRISPR). Latency reversal cure approaches have included the use of protein kinase C (PKC) modulators, histone deacetylase inhibitors (HDACis), bromodomain extra-terminal motif (BET) bromodomain inhibitors, and second mitochondrial-derived activator of caspases (SMAC) mimetics. Kill augmentation has been explored with broadly neutralizing antibodies (bnAbs), programmed cell death protein (PD-1) boosted cytotoxic lymphocytes (CTLs), chimeric antigen receptor T and NK cells (CAR-T/CAR-NKs), and bispecific/trispecific antibodies, while modified stem cells with CCR5-∆32 bone marrow transplants have been used to apparently cure HIV in a select few individuals.

Latently infected cells represent the major reservoir allowing persistence of the virus during ART. Therefore, most HIV cure efforts are directed towards eliminating this reservoir, with the goal of allowing PWH to stop ART without rebound occurring. Multiple cure strategies (Figure 4) have been explored within the field, including killing reservoir cells following virus reactivation, permanently silencing latent genomes, editing and inactivating the latent proviruses, and transplantation or gene-therapy approaches to create an HIV-resistant immune system.

### 5.1. Latency Reversal

The “activation–elimination” approach, otherwise known as “kick and kill” or “shock and kill”, is a cure strategy in which latently infected cells are induced to express viral proteins (kick) using latency reversal agents (LRAs), allowing the immune system or viral cytopathic effects to eliminate the infected cell (kill). Experimental approaches to reactivating the HIV provirus expression have included protein kinase C (PKC) modulators [101,135,136], second mitochondrial-derived activators of caspases (SMAC) mimetics [137], BET- bromodomain inhibitors [138], histone deacetylase inhibitors (HDACis) [139,140], and various other stimulation methods [7,141].

PKC modulators compete with endogenous ligand diacylglycerol (DAG) to bind to cytosolic PKC. Upon PKC modulator–PKC binding, a complex is formed and translocated into intracellular membranes which induces downstream signaling pathways that result in phosphorylation of IkB, enabling NF-kB-mediated transcription of the HIV provirus and triggering viral protein expression in latently infected cells [101,142]. Naturally-occurring PKC modulators, including bryostatin-1, and prostratin, can reverse HIV from latency in vitro and ex vivo [101,143,144]. Moreover, the design and synthesis of various analogs and slow-release prodrugs of naturally occurring PKC modulators have greatly improved the latency reversal capacity and in vivo tolerability of these compounds [100,101,135,145,146,147,148,149]. For example, a particularly potent bryostatin-1 analog was capable of inducing HIV expression in humanized BLT mice that were infected with HIV and ART-treated, leading to the death of latently infected cells and a reduction in the rebound-competent reservoir [100,101].

The non-canonical NF-kB pathway can also be exploited to reverse HIV latency, [150] and can be activated using SMAC mimetics [97,99,137], which block inhibitors of apoptosis proteins (IAPs) by antagonizing anti-apoptotic XIAP1, cIAP1, and cIAP2, promoting pro-apoptotic activity and enhancing tumor necrosis factor-dependent apoptosis [151]. As cIAP1 and cIAP2 degrade NF-kB-inducing kinase and block p100 processing into p52, SMAC mimetics allow NIK accumulation to eventually trigger the non-canonical pathway of NF-kB activation and the subsequent viral transcription [137].

Both BET-bromodomain inhibitors and HDACis represent LRA classes that target epigenetic modifications that silence expression, restricting HIV expression. BET-bromodomain inhibitors bind acetylated lysine residues (bromodomains) on BET family member BRD4, preventing competition with HIV Tat for downstream interaction with p-TEFB [138,152]. HDACis inhibit the deacetylation of chromatin, unwinding host DNA that may have integrated provirus and allowing access to transcription factors such as NF-kB. Historically, HDACis have been approved for T cell lymphoma treatment, but in vitro and in vivo studies have established that various HDACis, including vorinostat [139], romidepsin [153,154,155], and panobinostat [156], function as LRAs. HDACis have also been documented to synergize with other LRAs, including the PKC modulator bryostatin-1 [144] and SMAC mimetics [137], which may represent promising combinations for future in vivo studies.

Despite these important advances in our understanding of the pathways governing latency and approaches that can exploit those pathways, no LRA has yet been identified that can induce expression of all latent HIV in a safe and effective manner. Promising future efforts in this area are directed at creating improved individual or combination LRAs with better latency reversal properties and augmenting the killing of cells that have been reactivated to express viral proteins, to better eliminate these latently infected cells.

### 5.2. Kill Augmentation

To fully clear infected cells upon latency reversal, a few key factors must be considered when formulating an appropriate kill strategy. Depending on the immunological status of the individual prior to ART initiation and the length of time since ART was initiated, anti-HIV immune responses may be sub-optimal. This can be due to immunological damage that occurred during the chronic phase of HIV infection, or because anti-HIV immune responses have waned over time, due to insufficient HIV antigen exposure during ART. In untreated infection, CD8^+^ cytotoxic T lymphocytes (CTLs) can also eventually become exhausted, due to chronic exposure to HIV-1 antigens [157,158]. Moreover, the mutation-prone virus will eventually express epitopes that can escape CTL detection [71]. Finally, HIV-infected cells can downregulate HLA-A and HLA-B, while maintaining the expression of HLA-C and HLA-E, allowing for natural killer (NK) cell and CTL immune evasion [159]. Various methods have been employed to circumvent these barriers to killing HIV-infected cells that have been induced to express from latency.

NK cells have been investigated as a cell type that can be utilized to kill HIV-infected cells (including those reversed from latency) due to their ability to release cytotoxic granules, induce apoptosis via TNF-related apoptosis-inducing ligand (TRAIL) and via Fas/FasL, as well as promote antibody-dependent cellular cytotoxicity (ADCC) [160,161]. Activation of NK cells can be accelerated as a part of the “kick and kill” approach via administration of IL-15 [71,162]. NK cells can also be utilized to selectively target antigens via the attachment of the variable region of an antigen-specific antibody to the variable region of an anti-CD16 antibody (bispecific killer cell engagers (BiKEs)), which can attach to CD16 on the NK cell, thus allowing for NK cell-mediated killing of specific targets [163,164]. In the context of an HIV cure approach, BiKEs that target the gp41 stump of HIV env have been constructed [163]. These BiKEs were constructed by combining PWH-derived gp41 stump-specific antibodies, with the CD16-targeting variable region of a phage-derived antibody (NM3E2) [163]. The efficacy of gp41 stump-specific BiKEs were compared to gp41 stump-specific mAbs, and it was found that NK cell degranulation was 2.5–3.5-fold higher with the BiKEs approach than the mAb approach, quantified by CD107a expression [163]. Within a similar vein, chimeric antigen receptor (CAR) NK cells can be produced by modifying NK cells, for example to express CD4zeta, which can bind to HIV-1 gp120 and kill cells that are productively infected with HIV [165]. One limitation to these NK cell approaches, however, is that NK cells can be subject to immune exhaustion. Exhausted NK cells upregulate PD-1, which makes NK cells unable to degranulate, promote ADCC, and/or effectively produce cytokines [166,167].

Another proposed kill approach is through chimeric antigen receptor T cells (CAR-T Cells), which can specifically target infected cells expressing HIV-1 antigen. CAR T cells have proven effective in cancer treatment [168], and it has been speculated that arming CD8^+^ T cells with CARs directed towards HIV-1 proteins that are exposed on the surface of productively infected cells may improve their recognition and killing by the transgenic CD8^+^ cells. Example work involving HIV-specific CARs includes the creation of a second-generation D1D2CAR 4-1BB CAR T cell, which has been successfully used to transduce hematopoietic stem cells during the creation of BLT mice, where they can pass positive and negative selection during thymopoiesis [169]. The D1D2CAR 4-1BB CAR-T cell expresses a truncated CD4 molecule, which is missing the D3 and D4 subunits, allowing it to avoid IL-16 stimulation and engagement of its T cell receptor, and preventing it from serving as an entry receptor for HIV-1 [169]. In vivo data from D1D2CAR 4-1BB CAR-expressing BLT humanized mice have shown a delay in viral rebound after ART cessation, indicating that this kill approach has promise as a potential therapeutic. Future studies could combine the CAR-T cell approach with LRAs to evaluate their effect on reservoirs. Similar to NK cells, however, CAR-T cells also experience exhaustion after prolonged viral antigen exposure [169].

Immune checkpoint protein blockade (ICB) is an additional proposed method to improve the killing of cells productively infected with HIV. Upon prolonged immune activation resulting from persistent infection, CTLs display an exhausted phenotype, usually indicated by the upregulation of immune checkpoint proteins (ICPs) PD-1, LAG3, CTLA-4, TIGIT, CD160, CD244, and TIM3 [157,158]. ICBs can be used to counter the reduced immune response caused by ICPs, resuming the killing of infected cells. The blockade of PD-1 via PD ligand 1 (PD-L1)-specific antibodies has been shown to improve HIV-specific activity in exhausted CTLs [157]. Additionally, the ability for ICB to cause HIV-latency reversal in infected CD4^+^ T cells has been observed. Ex vivo stimulation of CD4^+^ T cells isolated from PWH with the PD-1 inhibitor pembrolizumab in conjunction with LRA showed an increase in viral RNA production [170]. Conversely, CD4^+^ T cells isolated from ART-unsuppressed and -suppressed individuals living with HIV, did not show an increase in viral production upon T cell receptor stimulation during PD-1 engagement [170]. SIV-infected ART-treated rhesus macaques given a CTLA-4 blockade showed a decrease in viral RNA in lymph nodes, as well as improved SIV-specific CD4^+^ and CD8^+^ T cell effector function [171].

Clinical trials with ICBs have shown varying levels of effectiveness, as treatment of PWH who also had cancer with anti-PD-1 did not show a consistent reduction in the viral reservoir, quantified by cell-associated HIV-DNA [157]. Additionally, transient HIV-1 latency reversal was observed in an HIV-infected, ART-suppressed patient who was given the immune checkpoint inhibitor of PD-1, nivolumab, while on ART [172]. This was observed in combination with an increase in HIV-specific IFNγ^+^ CD8^+^ cells, an increase in plasma IL-6, an increase in CD4^+^ and CD8^+^ cell counts, and a decrease in PD-1 in T cells. These findings suggest that nivolumab can improve the HIV-specific CD8-cell-mediated inflammatory response. However, despite these changes in the inflammatory state of the individual, there was little to no impact on reduction of the HIV reservoir. [172]. In an additional study, administration of ipilimumab (an inhibitor of CTLA-4) in a person with HIV, provided evidence of potential HIV latency reversal. Upon ipilimumab treatment, an increase in CD4^+^ T cells was observed, alongside an increase in cell-associated non-spliced HIV-1 RNA after each dose of ipilimumab. A subsequent decline in plasma HIV-1 RNA was later observed [173]. Additional evidence to support the potential efficacy of ICB on HIV latency reversal was observed in three patients who were given ICB infusions, and showed an increase in cell-associated non-spliced HIV-1 RNA. However, a decrease in HIV DNA was not observed [174]. Overall, these studies indicate that ICB may assist in HIV latency reversal and improve immune responses, but additional approaches will likely be needed to fully deplete the latent reservoir.

Broadly neutralizing antibodies (bNAbs) against HIV envelope proteins have been investigated as an additional kill approach to facilitate eliminating the viral reservoir. For example, 3BNC117, an experimental bNAb that is specific to the CD4 binding site on HIV Env, has been shown to delay viral rebound in a human analytical ART treatment interruption (ATI) study [175]. Additionally, VRC01 is another bNAb specific to the CD4 binding site on HIV-1 Env, but has shown only a modest delay in viral rebound after ART cessation in 24 human participants in two clinical trials, AIDS Clinical Trials Group A5340, and NIH 15-I-0140 [176]. Compared to historical controls, VRC01 caused a slight delay in viral rebound (median viral rebound time in the A5340 and NIH trials was 4 and 5.6 weeks, respectively) [176]. However, a combination approach of anti-HIV bNAbs has also been investigated with much more substantial effects. 3BNC117 and 10-1074, a bNAb that is specific to the base of the V3 loop of gp120, were both administered in humans two days before ART treatment interruption and resulted in a significant delay in viral rebound compared to bNAb monotherapy [177].

Another clinical trial investigated the use of 3BNC117 and 10-1074 co-therapy on ART-suppressed PWH, and assessed the time to viral rebound after ATI. Seven out of seven study participants that were given the bNAb treatment did not experience viral rebound before week 28 of the study (median time to viral rebound was 39.6 weeks), whereas six out of the seven study participants that were given the placebo treatment, experienced viral rebound before week 28 of the study (median time to viral rebound was 9.4 weeks) [178].

Further combinations of bNAbs have also been extended into the clinic, and have shown promising results. Five non-ART-suppressed PWH were given combinations of three bNAbs, PGDM1400 (HIV-1 V2-glycan-specific), PGT121 (V3-glycan-specific), and VRC07-523LS (CD4-binding-site specific) [179]. Individuals given a single infusion of a combination of all three bNAbs showed a sharp and rapid drop in viral load (median time to reach lowest level of viremia was 10 days). Viral rebound to pre-infusion levels occurred after a median of 20 days in all patients [179].

Similar to other cancers, chemotherapy-refractory chronic lymphocytic leukemia (CLL) involves an upregulation of factors involved in cell survival, allowing for the survival of cancerous cells [180,181]. To downregulate antiapoptotic pathways and facilitate killing of infected cells, BH3 (BCL-2 homology domain 3) mimetics, such as venetoclax, have been used with promising results and were approved for the treatment of chemotherapy-refractory chronic lymphocytic leukemia (CLL) [182,183]. In vitro characterization of HIV-infected cells has demonstrated a similar upregulation of antiapoptotic pathways, suggesting that the virus modulates these pathways to ensure the longevity of its host [184]. BH3 mimetics antagonize BCL-2 family pro-survival proteins, allowing cells to complete apoptotic pathways and more efficiently deplete HIV-infected cells [182]. In ex vivo CD4^+^ T cells from PWH, venetoclax successfully depleted intact and total HIV-1 DNA. Additionally, a prolonged 6-week treatment of venetoclax during ART treatment in humanized mice delayed viral rebound by up to 2 weeks after ART cessation [182]. It has been shown that a combination therapeutic approach of venetoclax with the myeloid cell leukemia sequence 1 (MCL-1) inhibitor S63845 can further delay viral rebound after ART cessation [182]. Inhibition of MCL-1, an anti-apoptotic protein and critical T cell regulator, by S63845, can inhibit antiapoptotic pathways that have been upregulated by HIV, and induce the depletion of infected cells [182,185].

Additional efforts to rejuvenate exhausted T cells have focused on the use of autophagy induction and the mTOR inhibitor rapamycin, which was found to reduce IFN-I signaling, a key driver of T cell exhaustion during an HIV infection [186]. Administration of rapamycin with ART resulted in decreased viral rebound upon ART cessation in BLT mice. Additionally, T cells analyzed from treated animals showed an improvement in functionality and downregulation of exhaustion markers, including PD-1 and TIM3 [186].

Taken together, these approaches (and other similar ones) may prove useful beyond augmenting the “kill” arm of “kick and kill” approaches to latency depletion. Strategies that provide long-term enhancements to HIV immunity, such as stem cell-based CAR-T gene therapies, or vectored production of bNAbs may also contribute to continued immune surveillance. This could help to rapidly eliminate HIV that emerges from rare latently infected cells that evaded cure efforts.

### 5.3. Stem Cell Gene Therapy

One potential approach for HIV cure is to replace the HIV target cells in vivo with those that are resistant to HIV infection. Without susceptible host cells, the virus would be unable to maintain a persistent infection, even in the absence of ART. This approach has proved successful in several individuals who were living with HIV and also required a bone marrow transplant for acute myeloid leukemia treatment. In these cases, the donors of the CD34^+^ hematopoietic stem cells used in the transplant each had a naturally occurring homozygous 32-bp deletion in the CCR5 gene (CCR5-∆32), resulting in a truncated CCR5 that is not expressed on the cell surface and thus unable to be used by R5-tropic HIV strains to enter cells [187]. These R5 strains are the most commonly transmitted variants and predominate throughout the presymptomatic phase of infection in most PWH. Several PWH including the “Berlin” [187,188], “Dusseldorf” [189], and “London” patients [190,191] received stem cell transplants from CCR5-∆32 donors. As a result of the treatment, the vast majority of HIV-infected cells present were cleared, while new immune cells were not susceptible to HIV infection, allowing HIV viral loads to remain undetectable even 13 years following transplant in one case [189].

While promising, chemotherapy and CCR5-∆32 bone marrow transplants are associated with many challenges, including high mortality risks and financial costs associated with the procedures, added complications that may result from graft-versus-host disease, and difficulties finding a matching CCR5-∆32 donor. Moreover, while a CCR5-∆32 transplantation may protect against the R5-tropic HIV, recipients remain vulnerable to reactivation of X4-tropic virus harbored prior to receiving their transplant [192]. However, these transplant successes represent important proofs of concept that HIV can be cured, and thus point the way to new approaches that could be more widely adopted. For example, a substantial research effort is currently directed towards developing gene therapy approaches that can disrupt CCR5 in an individual’s own stem cells before being transplanted back into the same person. Though allogeneic stem cell reinfusion has been demonstrated [193], developing autologous transplantation approaches may be necessary to avoid graft incompatibility and difficulties associated with finding appropriate HIV-resistant stem cell donors.

### 5.4. Block and Lock

A “block and lock” approach has also been considered for HIV cure, which, instead of inducing expression of reservoir cells, has the opposite aim of permanently silencing all latent proviruses. By interfering with HIV transcription, which occurs during reactivation of replication-competent reservoir cells and actively replicating viruses, both can be targeted with this strategy. This could be used to permanently silence latent proviruses and also remove the possibility of ongoing immune activation from HIV proteins produced by defective proviruses. HIV latency is highly dependent upon host transcription factor availability and is influenced by factors involved in chromatin and epigenetic conditions, transcriptional elongation, and integration site selection. Thus, there are multiple potential strategies that have been applied in block and lock approaches, including (but not limited to) didehydro-cortistatin A (dCA)-mediated Tat inhibition, Janus kinase (Jak)-STAT inhibitors, and bromodomain-containing protein 4 (BRD4) modulators [194].

An analog of naturally occurring steroidal alkaloid cortistatin A (CA), dCA binds to the TAR-binding domain of Tat, reducing P-TEFb-mediated transcriptional initiation and elongation [195]. These effects have been shown to be Tat-specific [196], and further observations of long-term dCA treatment have shown it may also block transcription through inducing repressive epigenetic changes such as increased nucleosome occupancy at the nuc-1 region of the 5′ LTR promoter and enhanced recruitment of BAF [196,197]. Importantly, dCA treatment in both BLT mice [197] and primary CD4^+^ T cells isolated from ART-suppressed patients [198] reduced and delayed viral rebound, establishing proof of concept for a potential functional HIV cure.

T cell homeostasis is, in part, regulated by cytokines that activate the Jak-STAT pathway, which is activated during HIV infection [199,200]. Originally approved for rheumatoid arthritis and myelofibrosis, Jak inhibitors ruxolitinib and tofacitinib have been assessed for their ability to inhibit HIV reactivation in cell lines and primary cells [201], as their anti-inflammatory properties may prevent HIV spread by reducing T cell activation. Both compounds demonstrated selective dose-dependent inhibition of HIV proliferation, indicating the potential for repurposing other similar anti-inflammatory drugs for blocking HIV reactivation.

BRD4 competes with Tat to bind to P-TEFb [152] and thus has been explored as an avenue for inhibiting HIV expression. Small molecule ZL0580, which binds to bromodomain 1 of BRD4, was identified as a compound for suppressing HIV transcription [202]. This is thought to occur through multiple mechanisms: inducing repressive chromatin structure at the LTR promoter and promoting BRD4 to CDK9 interactions, while reducing Tat-CDK9 interactions [202]. Taken together, this research has important implications for the epigenetic role of BRD4 in regulating HIV latency, and thus may represent another class of block and lock compounds.

One caveat to the “block and lock” approach is that replication-competent HIV genomes will remain in the host, as the goal of this strategy is not to eliminate the HIV genome, only silence it. It is difficult to predict with certainty whether these intact HIV proviruses could reactivate at some later date, for example, if transcriptional or epigenetic changes occur that relieve the block on expression. Currently, these agents are unable to induce long-term or permanent silencing of the HIV provirus unless continuously administered. However, they may be able to complement current ART by silencing intact or defective proviruses that contribute to chronic inflammation. Furthermore, the human genome has a large number of endogenous retroviruses that do not express under most circumstances, and driving latent HIV into complete dormancy in a similar fashion may be feasible, particularly if more readily reactivated latent HIV genomes are first purged by LRAs.

### 5.5. Direct Targeting of the HIV Provirus

Perhaps the most conceptually simple method for eliminating a latent virus that is encoded in host-cell chromosomes is direct editing of the viral sequence to remove or permanently inactivate it. There is a rapidly growing list of powerful molecular biology tools, which can directly edit genomic DNA in a sequence-specific manner, which have been exploited to achieve this goal. Early approaches to disrupting integrated HIV utilized evolved recombinases to excise the provirus [203]. More recent approaches have utilized zinc finger nucleases, transcription activator-like effector nucleases, or clustered regularly interspaced short palindromic repeat (CRISPR)/CRISPR- associated nuclease 9 (Cas9) for gene editing of the HIV provirus. In particular, CRISPR/Cas9 has risen to prominence due to advantages in limited off-target effects, the potential to be delivered by lentiviral and adeno-associated virus vectors [204], and reduced construction time in comparison to transcription activator-like nucleases and zinc finger nucleases. For example, CRISPR/Cas9 was used to genetically disrupt the HIV LTR, inhibiting active and latent provirus transcription [204]. Multiple additional studies have been conducted to assess whether removing integrated HIV or related retroviruses is possible through CRISPR/Cas9, with promising results in vitro [205,206,207] and in vivo [205,206,207].

Beyond direct targeting of the genome, CRISPR/Cas9 has also been used to reactivate latent HIV, where engineered single guide RNAs targeting various regions within the HIV LTR have been delivered in tandem with a deficient Cas9 combined with transcription activator domain-specific single guide RNAs [208,209,210]. Gene editing has also been used to modify host cells to make them refractory to HIV infection, such as CCR5 gene editing to inhibit the spread of R5-tropic HIV [211,212].

## 6. Conclusions

Developing a safe and scalable cure for HIV that could be extended to the 40 million PWH worldwide remains elusive. This is due to the complex mechanisms of latency establishment and maintenance, and the nature of the latent reservoir in vivo, which is composed of rare, long-lived, and systemically distributed cells. Though there are many complications associated with identifying and targeting reservoir cells, extensive work with isolated molecular biology systems, cell line models, primary human cells, and advanced in vivo animal models of HIV latency have identified several promising approaches for curing HIV. Importantly, these strategies are not mutually exclusive. An ultimate cure may be complex, for example, involving reactivation of the reservoir using LRAs, and the subsequent use of “block and lock” agents to permanently silence residual virus that is readily induced to express. Genetically augmented anti-HIV immune cells and molecules such as CAR-T cells and bNAbs might also be used to aid in the “kick and kill” process then assist long-term immune surveillance to rapidly eliminate any residual virus that evades initial cure efforts.

It should also be noted any HIV cure needs to provide significantly greater benefits and/or diminished side effects in comparison to lifelong ART, because with improved treatment, HIV has become a chronic but manageable condition for many. The kick and kill approach has the limitation that it must reactivate latent HIV in all reservoir cells to cure the infection, but might also be used to complement other cure strategies by depleting any residual reservoir cells that they fail to eliminate. Transplantations, which have proved efficacious in CCR5∆32 donor transplantation recipients, are limited by their complexity and by matching donor availability, and may require gene therapy to successfully scale. Block-and-lock approaches may also be useful as ART regimen supplements because they might prevent residual HIV protein expression that contributes to persistent immune activation and chronic inflammation in PWH on ART. However, they face the challenge of accomplishing permanent HIV provirus silencing in the absence of drugs. Finally, gene editing might offer a permanent solution through completely excising the HIV provirus or modifying susceptible cells to resist infection, but must successfully modify all reservoir cells while simultaneously avoiding off-target effects.

Though each of the approaches described here presents its own merits and challenges, a greater understanding of the mechanisms behind HIV latency maintenance and reservoir establishment, coupled with improvements in therapeutic strategies, show promise in eliminating persistent reservoirs and, ultimately, developing a cure for HIV.
